# Supplementary material for: Biosynthesis of Fatty Acid Derivatives by Recombinant Yarrowia lipolytica Containing MsexD2 and MsexD3 Desaturase Genes from Manduca sexta
Source: J Fungi (Basel). 2023 Jan 14;9(1):114. doi: 10.3390/jof9010114 (PMC9862095; doi:10.3390/jof9010114)
Supplement: Supplementary file 1 [file jof-09-00114-s001.zip › Supplementary material.pdf]

Gene name: MsexD2, Length: 1005 bp, Sequence:

CCCCGGATCCGGTCTCTAATGGCCCCCAACTTCGGTACCG
AGATGTCTGCCCACATCGACGCTGAGGAGTCCTACGAGAAGCTGA
TCCCTCCCCAGGCTGCTCCCCGAAAGTACAAGTACCTGTACGCCA
ACATGATCTACTTCGCCTACTGGCACATCGCTGGACTGTACGGTA
TCTACCTGGCTTTCACCTCTGCCAAGTGGGCTACCATCATTCTGG
CCTACCTGCTGTTCGTGGCTGGCGACATTGGAGTGACCGCTGGAG
CTCACCGACTGTGGGCTCACAAGTCTTACAAGGCTAAGCTGCCCC
TGCAGATCCTGCTGATGCTGTTCTCCACCATGGCCTTCCAGAACA
CCGTGATTACCTGGGTCAAGGACCACCGAATGCACCACAAGTACT
CTGACACCGACGCCGACCCCCACAACGCTACCCGAGGTTTCTTCT
ACTCCCACGTGGGCTGGCTGATGGTCAAGCGACACCCCGAGGCTA
TCAAGCGGGGCAAGTCTCTGGACATGTCCGACATCTACAACAACC
CCGTGCTGAAGTTCCAGAAGAAGTACGCCATCCCCCTGGTGACCA
CCATTGCTTTCGTCCTGCCCACCATCATTCCCATGTACTTCTGGG
ACGAGTCTTTCAACGTCGCCTGGCACATGACCATGCTGAAGTACA
TCTTCGGACTGAACGCCGCTTTCCTGGTGAACTCTGTCGCCCACA
TGTGGGGTTACAAGCCCTACGACAAGAACATCGCTCCCACCCAGT
CCTACATTGCCACCTTCGCTACCCTGGGCGAGGGATTCCACAACT
ACCACCACGTGTTCCCTTGGGACTACCGAGCTTCTGAGCTGGGCG
ACAACTACCTGAACTTCACCACCAAGTTCATCGACTTCTTCGCCT
GGATTGGATGGGCTTACGACCTGAAGGCTGCTCCTGAGGACCTGG
TCCAGAAGCGAATTGAGCGAACCGGTGACGGCACCAAGCTGTAGT
CTAAGAGACCCCTAGGCCCC

Gene name: MsexD3, Length: 1065 bp, Sequence:

CCCCGGATCCGGTCTCTAATGGCCCCCAACTTCGGAAACG
AGGTGTCTTCCCCCATCGTCGCTGAGGAGTCTTACGAGAAGCTGA
TCCCTCCCCAGGCTGCTCCCCGAAAGTACAAGTACCTGTACGCCA
ACATGATCTACTTCACCTACTGGCACATTGCCGGCCTGTACGGAA
TCTACCTGGCTATTACCACCGCCAAGTGGGCTACCATCATTCTGG
CCTACCTGCTGTTCGTGGCTGGAGAGATCGGCATTACCGCTGGTG
CTCACCGACTGTGGGCTCACAAGTCCTACAAGGCTAAGCTGCCCC
TGCAGATCCTGCTGATGCTGTTCAACTCTACCGCCTTCCAGAACT
CCGTGATTACCTGGGTCAAGGACCACCGAATGCACCACAAGTACT
CTGACACCGACGCCGACCCCCACAACGCTACCCGAGGATTCTTCT
ACTCCCACGTGGGTTGGCTGATGGTCAAGCGACACCCCGAGGCTA
TCAAGCGGGGCAAGTCTCTGGACATGTCCGACATCTACAACAACC
CCGTGCTGAAGTTCCAGAAGAAGTACGCCATCCCCCTGATTACCA
CCGTGGCTTTCGTCCTGCCCACCATCATTCCCATGTACTTCTGGG
ACGAGTCTTTCAACGTCGCCTGGCACATGACCATGCTGCGATACA
TCATTAACCTGAACACCATCTTCCTGGTGAACTCCGTCGCCCACA
TGTGGGGCTACAAGCCCTACGACAAGAACATCGCTCCCACCCAGA
ACTACATTGCCACCTTCGCTACCCTGGGCGAGGGATTCCACAACT
ACCACCACGCCTTCCCCTGGGACTACCGAGCTTCTGAGCTGGGCA
ACAACTACCTGAACCTGACCACCAAGTTCATCGACTTCTTCGCCT
GGATTGGATGGGCTTACGACCTGAAGACCGTCCCCGAGGACCTGC
TGCAGAAGCGAATGGAGCGAACCGGTGACGGCACCAACCTGTGGG

GACGAGGTGACAAGAACATGAAGAAGGACTACGTGAAGTCCACCG
ACGTCCACGAGTAGTCTAAGAGACCCCTAGGCCCC

Figure S1 Gene sequences of *MsexD2* and *MsexD3*

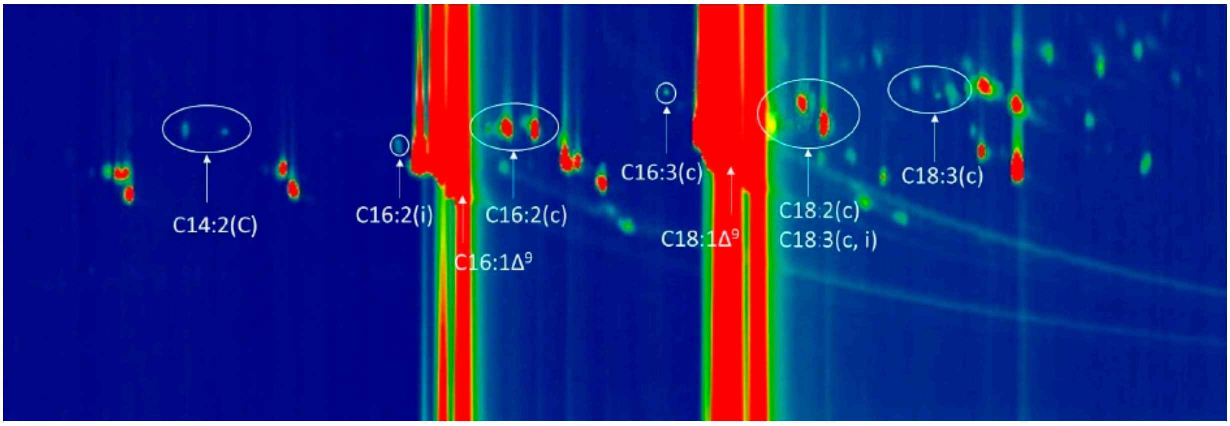


Figure S2: GCxGC-MS chromatogram of yeast sample 7084 (co-cultured with C16:2$\Delta^{10,12}$-Me) with description and location of new fatty acids. (c) – conjugated double bonds, (i) – isolated double bonds, (c, i) – one conjugated and one isolated double bond. Oleic and palmitoleic FAs are highlighted for better orientation in chromatogram.
